# The impacts of local networks on subsistence resilience and biodiversity in a low-lying Moluccan reef system between 1600 and the present

**DOI:** 10.1007/s13280-018-1091-2

**Published:** 2018-09-05

**Authors:** Roy Ellen

**Affiliations:** School of Anthropology and Conservation, Centre for Biocultural Diversity, University of Kent, Marlowe Building, Canterbury, Kent CT2 7NR UK

**Keywords:** Biodiversity change, Central places, Environmental risk, Moluccas, Reefs, Trading networks

## Abstract

Using field data for the 1980s and historic material, I show how the central places of networks crucial for regional and long-distance trades in the Moluccas between 1600 and the present were often environmentally vulnerable volcanic islands and low-lying reefs. After reviewing existing data on hazards, and evaluating the evidence for erosion and degradation, I suggest how resilience has been historically achieved through social and material exchanges between islands, accommodating the consequences of specific perturbations. Re-interpretation of published data shows how inter-island trade has re-organised patterns of biological interaction spatially and over the long term, helping us assess whether, in the face of climate-change effects, such areas are zones of robustness or of potential fragility.

## Introduction

The central places of networks that we know to have been crucial in regional and long-distance trades in the Moluccas between 1600 and the present were often environmentally vulnerable volcanic islands and low-lying reefs. Nevertheless, they have persisted as important hubs over many centuries, riding periodic denudations and coping with physical hazards and biodiversity asymmetries. This paper reviews the evidence we have for hazards in such locations, and for erosion and degradation. The analysis builds upon and re-evaluates the findings of Ellen’s *On the Edge of the Banda Zone* (2003), based on fieldwork conducted between 1980 and 1986. Other major historical studies relevant to the articulation of international and local trade in this area include Meilink-Roelofz (1962), Knaap (1985), and Andaya (1991, 1993), and their relevant findings are incorporated in the *Banda Zone* book. Most of the data were not originally published with a view to their relevance to the literature on environmental-change impacts on small islands ( e.g. Kelman 2017), but are here re-assessed in relation to current thinking. Specifically, I show how the resilience of local systems was

1. embedded through progressive physical modification of landforms and biota;
2. effected through social exchange between components of a system that were individually resource poor;
3. reflected in the ability of human inhabitants to move to accommodate the consequences of perturbations; and has
4. likely mitigated climate-change effects on both human settlement and biodiversity.

Studies of trade and biodiversity decline in Indonesia have focussed on commercial extraction of forest or marine resources (e.g. Edwards and Nash 1992; Sodhi et al. 2004), rather than on how trade has re-organised patterns of biocultural interaction locally, spatially and over the long term. My concern is less with decline in biological richness due to trade, than with changes arising from both global temperature rise and local environmental manipulation, and also with the associated structural re-alignment in the distribution of resources in a particular area.

## Theoretical framework, methods and materials

The research described is framed within the paradigm of historical ecology, informed by systems-oriented environmental anthropology. It draws on ethnographic fieldwork conducted in 1981 and 1986 in East Seram subdistrict. Interviews, conducted in Indonesian directly by the author, were sometimes pre-arranged and otherwise opportunistic. They varied between informal conversations and semi-structured formats, following the accepted conventions of intensive qualitative research, involving an estimated 26 individuals between January and March 1981, and 97 individuals between March and June 1986. The interviews yielding data relevant to this paper, including personal recollection and oral history data on seismic activity, flooding and climate-related events, engaged mainly local government officials, fishermen, boat owners and traders (both male and female), living mainly in the settlements of Geser, Keffing, Kilwaru, Seram Laut, Gorogos and Kidang. Most interviews were with individuals, but a few were with groups. The surveys involved systematic documentation of relevant landscape features, vegetation cover (including anthropic vegetation) and local knowledge of that vegetation, physical inspection of flooded areas or areas subject to flooding, and of mangrove depletion and the condition of coral, together with physical infrastructure built and maintained to resist flooding and erosion. All the obtained data were supported by mapping and photography. The methods used in the trading network analysis are reported in Ellen (2003, pp. 178–211). The historical work (mainly 1984) involved systematic checking of records for east Seram in the Dutch East India Company (VOC) archives (for the period before 1795), and in colonial archives (from 1796 onwards, mainly the ‘mail reports’) located in The Hague; and library research in the Netherlands and the UK. I have also used data on seismic activity provided by the Global Seismology Unit in Edinburgh, records lodged in the Geser office of the Indonesian meteorological service, data collected by Hermien Soselisa during fieldwork on Gorogos in 1993, and secondary sources on ecology, history and ethnography. The start year of 1600 for the timeline marks a convenient date after which the historical records are sufficiently robust to allow plausible reconstruction of trade patterns, local environmental histories, island life and interconnections.

## Local trade in spatial and historical context

**Fig. 1:**
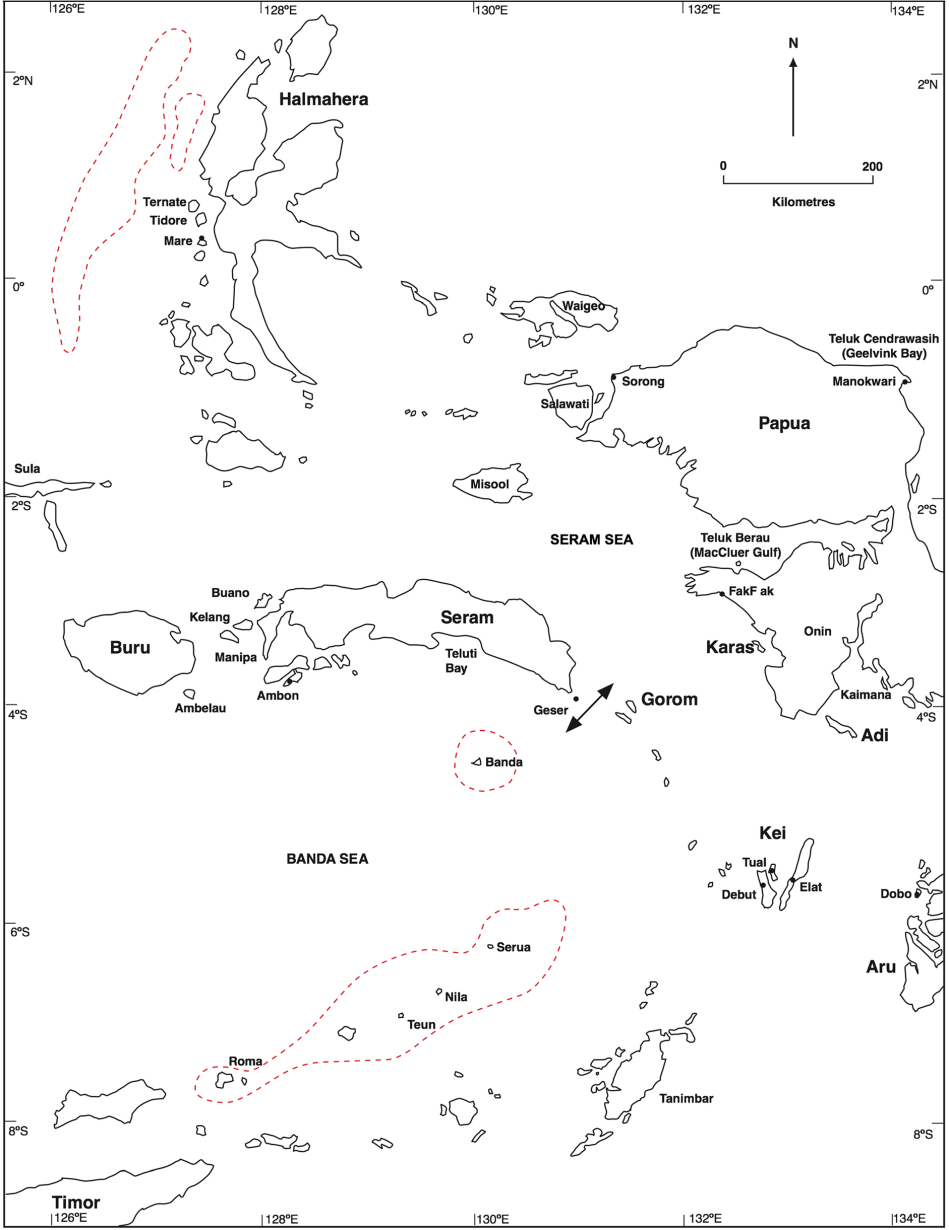
The Moluccan islands showing areas of highest seismic activity (marked by broken line) between 1899 and 1978 in relation to historic hubs of production and exchange, and indicating the Geser–Gorom corridor (double-headed arrow). *Source* Earthquake Data File 1981, of the Global Seismology unit of the Institute of Geological Sciences in Edinburgh; based on 4542 events for quadrat longitude 125.0W to 135.0E and latitude 5.0N to − 10.0S

The context of this paper is the interaction between local and long-distance trade, well documented from 1600 onwards. The small islands offshore larger islands in the north and central Moluccas (Fig. 1) have historically subsisted by trading fish for sago and other food and resources supplied by a larger island ‘mainland’. This facilitated the development of their role in international trade as either producers of spices or marine produce, and as hubs for the trade in products coming from New Guinea and the peripheral Moluccan islands, and for rice and manufactured goods coming from the West and North.

These offshore islands were either volcanic or uplifted coral reefs. The volcanic islands tended to be centres for clove (Ternate and Tidore) or nutmeg (Banda) production. The two spices were essentially monocropped, and by the colonial period the producing islands had only limited space for homegardens, fruit groves and physical settlements. Sustainability was enhanced by intercropping other trees, such as *Canarium* on Banda. By comparison, the trading centres on the reefs of the Outer Banda Arc were even smaller islands with fewer terrestrial resources, although with access to rich fishing grounds, and craft skills producing items circulating throughout the central and southeastern Moluccas.

These islands have limited and fragile vegetation, and might be characterised as biodiversity cool spots, whether volcanic (e.g. Paulay 1994) or coralline (see e.g. Thaman 2008). Like many ecosystems experiencing long-term anthropogenic impacts, it is difficult to determine what the ‘natural’ vegetation might have been (e.g. Kareiva et al. 2007). Nevertheless, taking all flora into account—crops, introduced weeds and endemic species—the islands off southeast Seram are especially biodiversity poor, although we cannot know the extent to which pre-human vegetation was replaced or eroded. Anthropic intensification accompanying European intervention exacerbated the decline of terrestrial biodiversity, but also increased pan-global invasives and introduced species from the Americas. Loss of vegetal diversity has been offset by the deliberate planting of trees and small homegardens. As the trading—and latterly administrative—role of the small islands grew, so did population and density of physical settlement, in places leading to ‘urbanised atolls’ (see e.g. Landsat images of Kiltai (for 2015), and Seram Rei (for 2016 ((c) DigitalGlobe, accessed via Google Earth 6 January and 7 February 2016).

The main historic threat to trading centres and biodiversity on volcanic islands has been volcanic eruption and other seismic activity (e.g. Torrence et al. 2000), well reflected in the Moluccan literature (Ellen 1987, pp. 48–50; Harris and Major 2016). Ternate, Tidore and the Banda archipelago have been centres of repeated events, including high-magnitude events of 7.75 or greater, with a wide radius, and causing a high intensity of damage. By comparison, the coralline islands of southeast Seram—although on a plate boundary—experience relatively low levels of direct seismicity. While this does not exclude serious mega-events over the long term, these have been infrequent (Liu and Harris 2014). Only two small events are recorded for the Geser–Gorom corridor for the period 1899–1978, of less than 60 and 300 kms depth and with radii of 5.5 or less. This contrast is shown in Fig. 1, based on the Earthquake Data File 1981 of the Global Seismology unit of the Institute of Geological Sciences in Edinburgh (see Harris and Major 2016 for data on earlier events). For most of this period, human structures were relatively lightweight, their collapse doing little damage and easily reconstructed. The main damage and human deaths would have come from consequent tsunamis.

## Archipelagic southeast Seram and its micro-climate

**Fig. 2:**
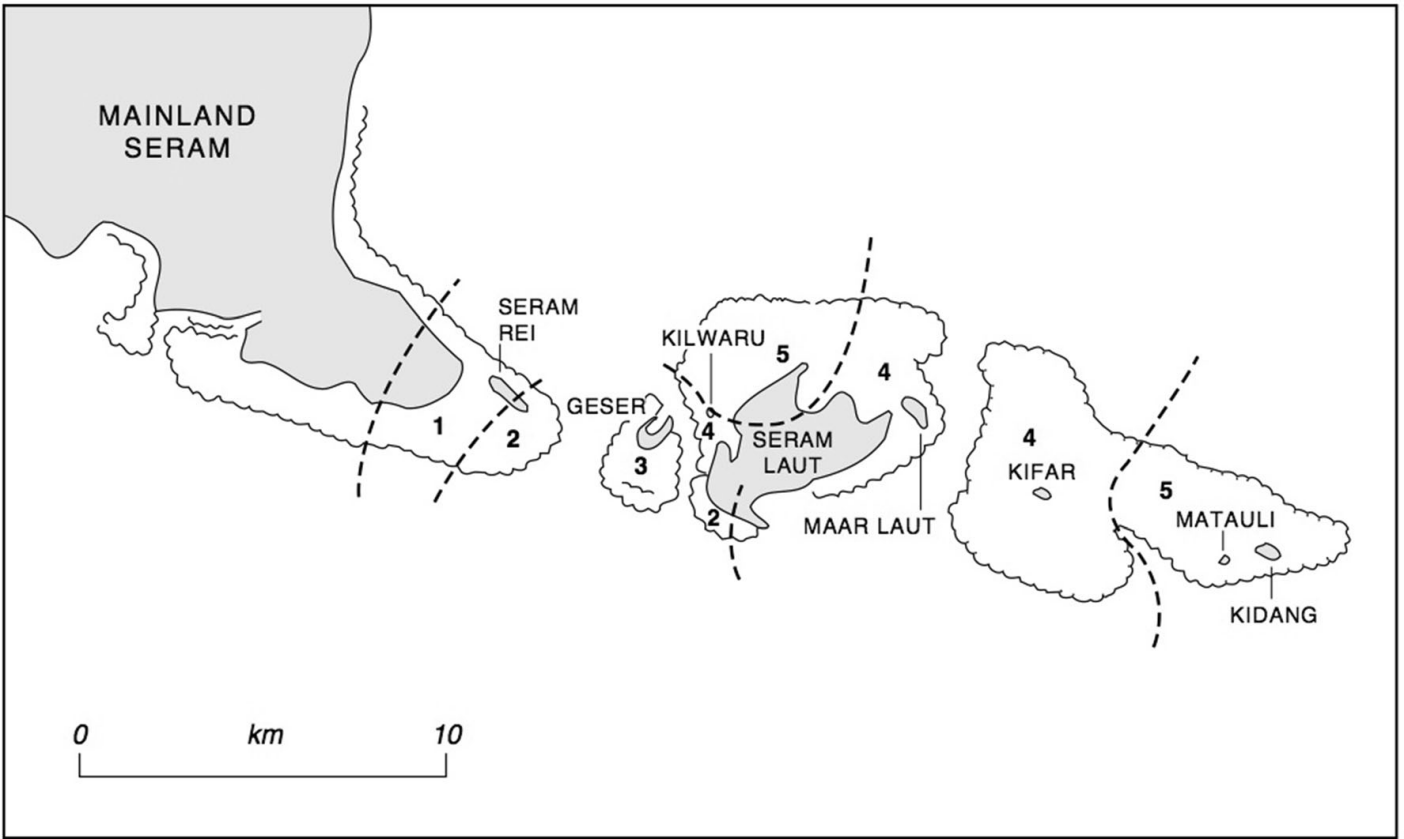
The Geser archipelago and nearby mainland Seram. Reef ownership in 1986 according to traditional political domains is shown as follows: 1 Keffing, 2 Kelu, 3 Geser, 4 Kilwaru, 5 Kiltai. *Source* Ellen 2003, p. 43

I focus on the Geser network that incorporates part of the mainland and small islands off the southeast tip of Seram (Fig. 2), not well described in the biodiversity literature for either coral or mangrove (Tomascik et al. 1997). This zone is united by a single language—Geser–Gorom or Southeast Seram Littoral (Loski and Loski 1989)—the distribution of which reflects the importance of regional and global trades over many centuries, and a socially and culturally coherent space with an identifiable internal structure. However, in order to understand the history of the littoral of Southeast Seram, and its social and economic character, it is also necessary to place it within a wider sphere of interaction extending to the New Guinea coast, and to the Kei and Aru archipelagos. This larger whole was historically articulated with global trade westwards through Banda (Ellen 2003).

The weather pattern is complex, although the main micro-climatic parameters are clear enough (Table 1). On Geser, rainfall peaks at 250 mm in May–June, with a 54 mm minimum in October. There are no clear rainy and dry seasons, precipitation varying from month-to-month outside the May–June peak, with a low rainfall trough in April. The East monsoon brings rain, rough seas and high tides during April–September; while the West monsoon during November–March brings drier weather, calm seas, clear water and low tides. There are cyclones between December and February. The highest tides are March–April, between the East and the West monsoons. On 29 April 1986 I recorded a high tide on Geser lagoon, where the difference between high and low tide was 1.5 m.

**Table 1:** Meteoreological data for Geser 1985. *Source* Departemen Perhubungan, Badan Meteorologi dan Geofisika, Balai Wilayah IV, Stasiun Meteorologi Geser. Data Klimatologi 1985

Month	Average (Temp C)	Rain mm	Wind speed-average (knt)	Wind direction (°)	Max speed (knt)
J	27.7	141	7	230	20
F	28.1	96	5	250	35
M	28.3	142	5	360	24
A	28.0	66	6	270	20
M	27.4	219	6	090	20
J	26.5	251	11	120	20
J	26.0	150	12	140	25
A	25.7	200	11	130	20
S	26.5	180	10	170	22
O	27.8	54	7	180	20
N	28.5	154	5	180	20
D	28.2	92	6	230	20

## Geomorphology and historical ecology

Of the wider system defined linguistically, and through trading activity focussed on measureable flows and networks (Ellen 2003, pp. 176–211), I here examine the central area encompassing the Seram Laut archipelago and the adjacent mainland, including the main traditional shipping lane connecting the Banda and Seram seas: the Geser–Gorom corridor (Fig. 2). Ecologically, seven major biotopes are distributed unevenly across the corridor: (i) rain forest, (ii) a coastal cline, (iii) a cultivated zone, (iv) mangrove, (v) reef (both fringing and barrier), (vi) coral atolls and cays and (vii) open sea. The inhabited reefs, cays and atolls that I discuss are—moving from West to East: Seram Rei, Geser, Kiltai (also called Kilwaru), Maar Laut, Kifar, Kidang, and Garogos (not shown on the map). This patterning, crucial for understanding how the area functions as a social system, can be further elaborated as follows:

1. *Rain forest* Restricted to mainland Seram, overlying sedimentary rocks at high altitudes and neogenous coralline deposits along the coast. Mature forest is extensively modified, with high densities of useful trees, such as *Canarium*, and including 814 km^2^ of semi-managed sago (*Metroxylon sagu*) swamp.
2. *Coastal zone* Roughly banded with *Canavalia rosea* along the herbaceous edge of the sandy littoral. *Nypa fruticans* is found in places, and inland a formation dominated by *Barringtonia racemosa* and *B. speciosa*. Behind rocky coast, there is a mixture of savanna bush, including secondary growth of *Macaranga* and *Melaleuca cajuputi*. Typical also are sea grasses, such as *Enhalus acoroides* and *Thalassia hemprichii*. Apart from the mainland, this structure is only clear on Seram Laut, a hilly limestone island rising to 90 m asl.
3. *Mangrove* In 1986, mangrove (*akat*) mainly constituted an extensive block at the eastern tip of mainland Seram (Fig. 3a), while Fig. 3b indicates its shape in the late seventeenth century. While comparing between the maps, it is possible to identify continuities in the distribution of mangrove over a period of several centuries, together with the location of human settlements. There is also mangrove along parts of Seram Laut, the Geser lagoon and Seram Rei. Mangrove around Seram Rei has eroded since the time the earlier map was made, giving rise to a much smaller and clearly defined island. *Avicennia officianalis* and *Sonneratia* occurs along the seaward edge, various *Rhizophora* in the interior, and *Bruguiera* landward.

**Fig. 3:**
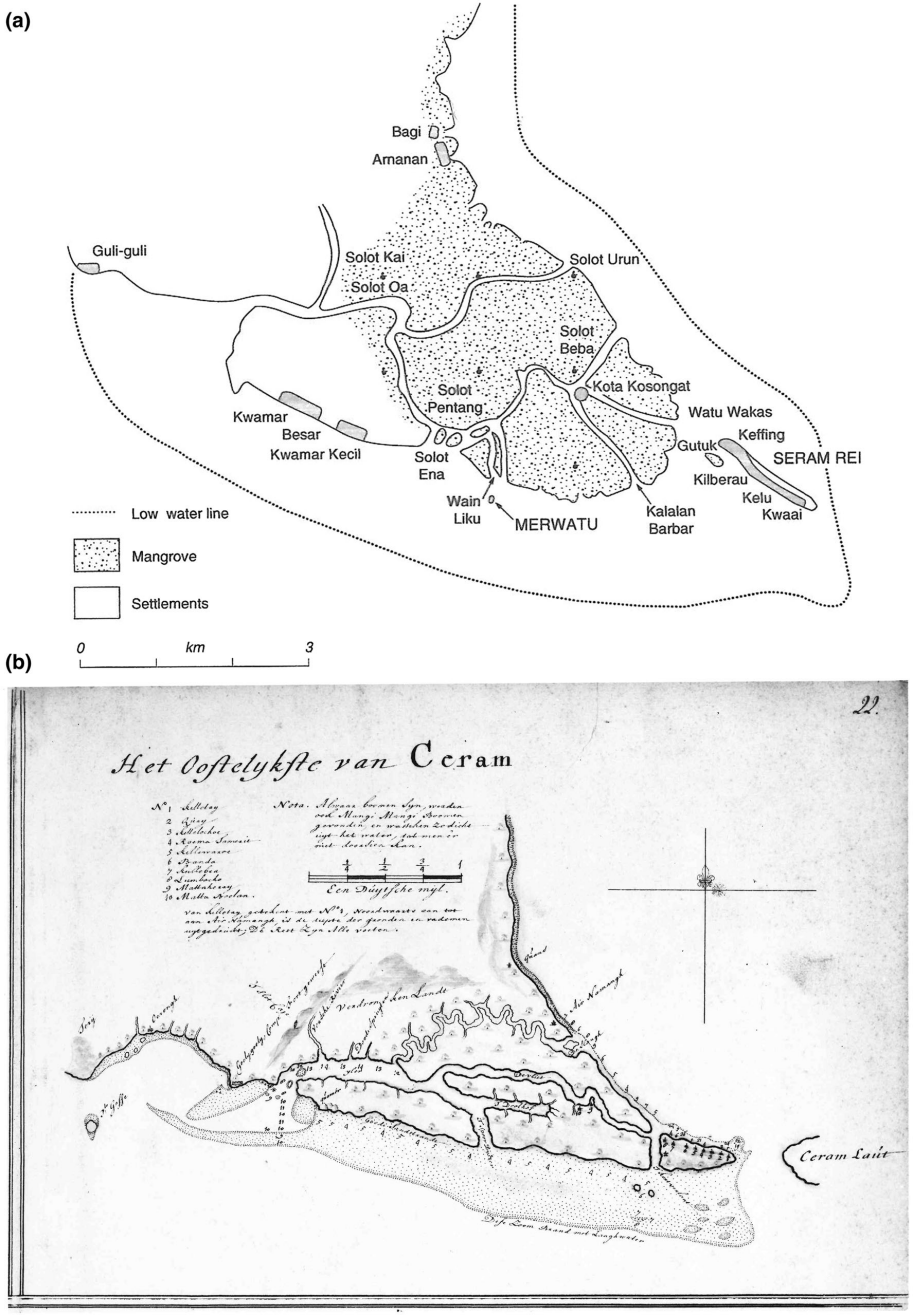
**a** The southeast Seram mangrove area as it was in 1986. **b** Detailed chart of the southeastern corner of Seram showing inland waters through the mangroves after 1688. The Solot Kai fort is marked 1 on the map. *Source***a** Ellen 2003, p. 26; and **b** Rijksarchief VEL 483, *Kaart van het oostelykste gedeelte van Ceram*
4. *Coral* Despite its significance in the lives of Serammers, coral is not recognised locally as a single lexically defined folk domain. Coral forming the base of islands is called *watu lian* (lit. ‘spikey stone’). Most other hard corals are known as *lodar* (‘karang’ in Malay), although some are neither *watu* nor *lodar*, such as *sisifi*. Seram Laut has coral walls dropping into the sea, often 50 m high, while most islands are surrounded by steep fringing reefs (*sakaru*). In addition are black, fine soft corals, conceptualised as living things. Unvegetated cays of broken dead coral thrown up from the reefs (*bas*, or ‘tanusang’ in Malay) are also a notable feature. The soil of smaller vegetated cays, such as Gorogos, is fragile, with flora—other than the usual pioneering species and those deliberately cultivated—being limited. This includes various species of *Pandanus*, stunted *Cocos nucifera, Casuarina equisetifolia*, *Plumeria obtusa, Bougainvillea*, the occasional date palm and scrub.

## Flooding risk, potable water and sea-defences

Geomorphology and micro-climate suggest that the small islands of the Geser–Gorom corridor are vulnerable to environmental hazards. However, given the regional pattern of seismic activity and physical geography, Geser is at reduced risk from local tsunamis. However, high tides (*tonu*) are a seasonal hazard (although rainwater flooding is virtually unknown). The high tide in Geser lagoon during the 1980s regularly flooded paths and edges of settled land. There was severe flooding in Geser in 1976, but only in the areas called Lomin and Kilwaru. Kiltai is flooded by rising spring tides once a year, destroying trees and other plants and polluting drinking water for 2–3 days. The severity of tidal flooding on Kiltai was reported by van der Crab (1864, p. 541), noting that people temporarily moved to Seram Laut at such times. Although high tides pose a periodic predictable hazard, low tides make it possible to walk between Kiltai and Seram Laut, and between Seram Rei and mainland Seram. Rosenberg (1875, p. 132) reported in the 1860s, as Bik (1928 [1824], p. 16) had before him, that it was very easy to walk from Keffing (on Seram Rei) to the east coast of the mainland. If there had been any significant rise in sea-level over the last few decades prior to fieldwork we might expect this to have become more difficult.

Soselisa (2004, p. 18) reported 12 wells on the inhabited cay of Garogos in 1993, two dry and all more-or-less brackish, with only two used for drinking water. Rainwater was collected to supplement this shortage, a pattern on other small islands in 1981 and 1986. The drinking water from wells within the domains of Kiltai, Seram Rei and Geser is brackish and its potability reduced even further during floods. At such times it is permitted to collect water from other domains, such as Kilwaru (also on Kiltai island). In addition to seawater contamination, the absence of creeks means that in the driest months, water sources disappear completely, as on Seram Laut. Collecting rainwater runoff helps reduce this risk. Despite frequency of flooding, drinking water availability has been pretty constant for 150 years at least. Wallace (1962 [1869], p. 288), for example, noted in 1860 that Kiltai water was of good quality.

**Fig. 4:**
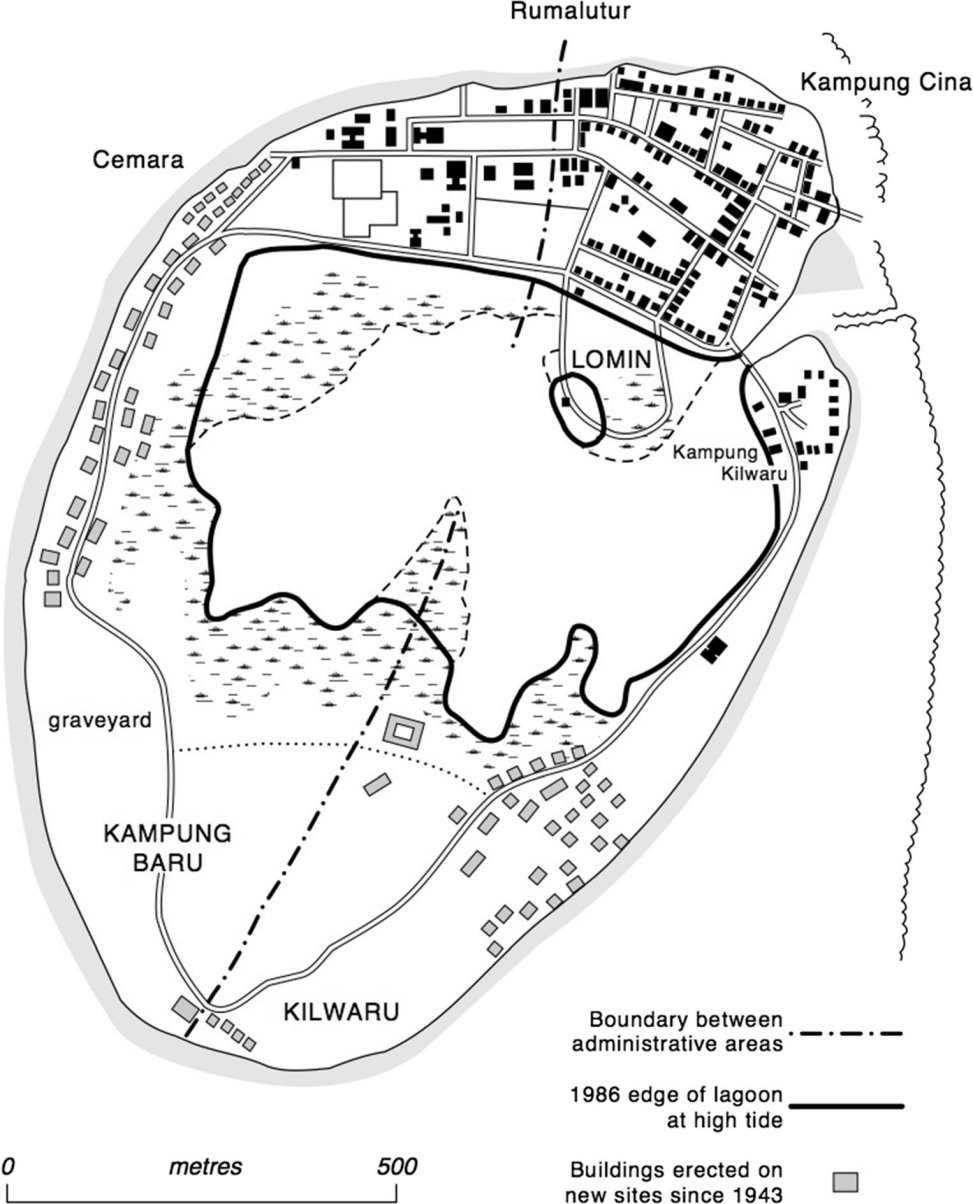
Geser Island, circa 1943. *Source* Ellen 2003, p. 117 (based on Map 9, Netherlands Forces Intelligence Service, No. 216a)

Human depletion and management of mangrove, and reconstruction of landforms using coral, have altered local topography over many centuries, in ways that are socially and ecologically significant. Kiltai, a low flat islet of coral and sand, has long been covered in dwellings, and its surface and edges reinforced with additional coral. A common sight in the Geser area continues to be collection by canoe of coral (particularly rock-like brain coral such as *Goniastrea*) from below the water level in shallow areas of sea, and its transport to where needed for house building, and to strengthen sea walls. Harbours and piers (*kotar*), such as those on Kiltai, and most visually arresting, *lutur* or stone fish traps, have been built of coral in numerous places. Coral is also a source of burnt lime, for cement and rendering, and for betel-chewing. The Geser atoll, only a few metres above sea-level, is composed of loose sand and coral, and its lagoon (or *lomin*, literally ‘inside’ or ‘full’) once contained an island, marked on colonial maps (Fig. 4). Over the years this island has eroded due to extraction of coral and sand to extend the village edge further into the lagoon, so increasing the contiguous land area available for habitation. The long-term effects of such engineering have been to raise the level of the land.

Coral has been used for other structures, such as walls surrounding compounds of local rulers, and forts for protection against rival domains, and later from the Dutch. Late eighteenth century coral forts are reported for Ondor and Kataloka on Gorom (Bik 1928 [1824], pp. 22–23), while Doren (1857, p. 314) notes that in 1659 a fort had been built east of Solot Kai (Fig. 3b) by the inhabitants of Guli-guli. Remains near Seram Rei were in 1986 recognised as belonging to Rumakat (lit. ‘house of the mangrove’), the main descent group in Kwamar, and alleged to date from the time of wars with Tidore. Called Kota Kosongat, this place in the mangrove today consists of no more than several piles of coral and some rocks showing evidence of human modification. Although Tidore continued to exercise influence over the coast in the *Tutu tolu* alliance area probably into the eighteenth century, Keffing (on Seram Rei) effectively prevailed (Andaya 1991, 1993a; Ellen 1993).

In addition to coral as a building material, there was timber. Wooden groynes afforded some defence against incursions from the sea. Moreover, traditional dwellings *(ruma loi*–*loi*, or houses raised on stilts) were previously a solution to the problem of high tide flooding. Few of these now remain. Coral limestone and cement structures replacing older housing are more vulnerable to flooding without appropriate protection. A major quake would have more impact on the Geser archipelago today than, say, 150 years ago, due to population growth, reliance on more permanent infrastructure and dependence on commodities such as kerosene, along with sea-level rise and reef erosion. However, the capacity to seek refuge and move quickly between islands, and to import food supplies from the Seram mainland and other parts of the archipelago, is still maintained.

## Subsistence, demography and patterns of settlement

Reef and lagoon topography is culturally dominant in archipelagic Southeast Seram, *bas* (reef) and *lomin* (lagoon) being conceptually complementary, in much the same way as mountain and sea, and heaven and earth in other cosmographies. However, poor or entirely absent soils, and concentration of settlement on restricted and precarious sites, has modified the environment, often in unusual ways. We have examined technical solutions to flooding and absence of potable water, but the limiting factor for plant cultivation is not water so much as space and the capacity of the substrate to establish soil. Simple gardens and fruit trees are kept by a few people (for example at the two extremities of the long thin cay of Garogos), some are adjacent houses and others on uninhabited islands. Soselisa (2004, pp. 20, 27) reports that in 1993 Garogos gardens contained taro, papaya, pumpkin, squash, watermelon, and that those with access through kinship to the more fertile islet of Kidang could obtain banana, cassava, mango and chilli. Garogos also supported edible wild plants: pan-global pioneering xerophytic succulents, such as shoreline purslane *Sesuvium portulucastrum* and the chickenweed *Portulaca quadrifida* (an invasive mat-forming species). But most non-marine food, construction materials, such as timber and sago palm thatch, has to be obtained from mainland Seram, Seram Laut or the large islands of the Gorom group, through barter, cash trade, kinship links, customary harvest leasehold and rights of direct ownership.

Despite physical constraints, populations on these small coral islands are high, dense and durable over the long term (Table 2). There is little evidence of periodic destruction, or even temporary abandonment. Soselisa provides no evidence in her work on Garogos. Indeed, Garogos (and Kifar), apparently uninhabited in 1850 (Bosscher 1855, p. 38), had a population of 126 in 1882 (Riedel 1886, p. 148) and 217 in 1993. Garogos and Kifar therefore had a growing population over a 100-year period, although, no doubt, with some fluctuations. As remarkable, is the population of Geser atoll, reported at 100 in 1862, before it overtook Kiltai as the preferred base for incoming traders, reaching 2216 by 1980.

**Table 2:** Island surface area, populations and density for small islands in the Geser–Gorom corridor. Some figures are estimated where published data are for local domains extending over several islands

	Area (ha)^a^	Population					Density
		1862^e^	1882^b^	1978^c^	1980–1981^a^	1993–2015	(per ha)^a^
Centres							
Geser	49.60	100	306	2178	2216		44.67
Seram Rei	39.88	570	527	417	485	725 (2015)^f^	18.18
Kiltai	5.23	270	568	520		470 (2015)^f^	89.87
Outliers							
Garogos	22.68}	126				217 (1993)^d^	9.57
Kifar	18.36}						
Kidang	11.76			40		80 (2015)^f^	6.80
Maar Laut	9.75			90		40 (2015)^f^	4.10

^a^Statistik Tahunan, Kecamatan Seram Timur Dalam Angka 1982: Kantor Wilayah Seram Timur

^b^Riedel 1886, p. 148

^c^Statistik Tahunan, Kecamatan Seram Timur Dalam Angka 1978: Kantor Wilayah Seram Timur

^d^Soselisa 2004, p. 22

^e^Van der Crab 1862, pp. 60–61

^f^Calculations based on number of households (Landsat GoogleEarth data) multiplied by 5 (the average household composition for Garogos provided by Soselisa 2004)

**Fig. 5:**
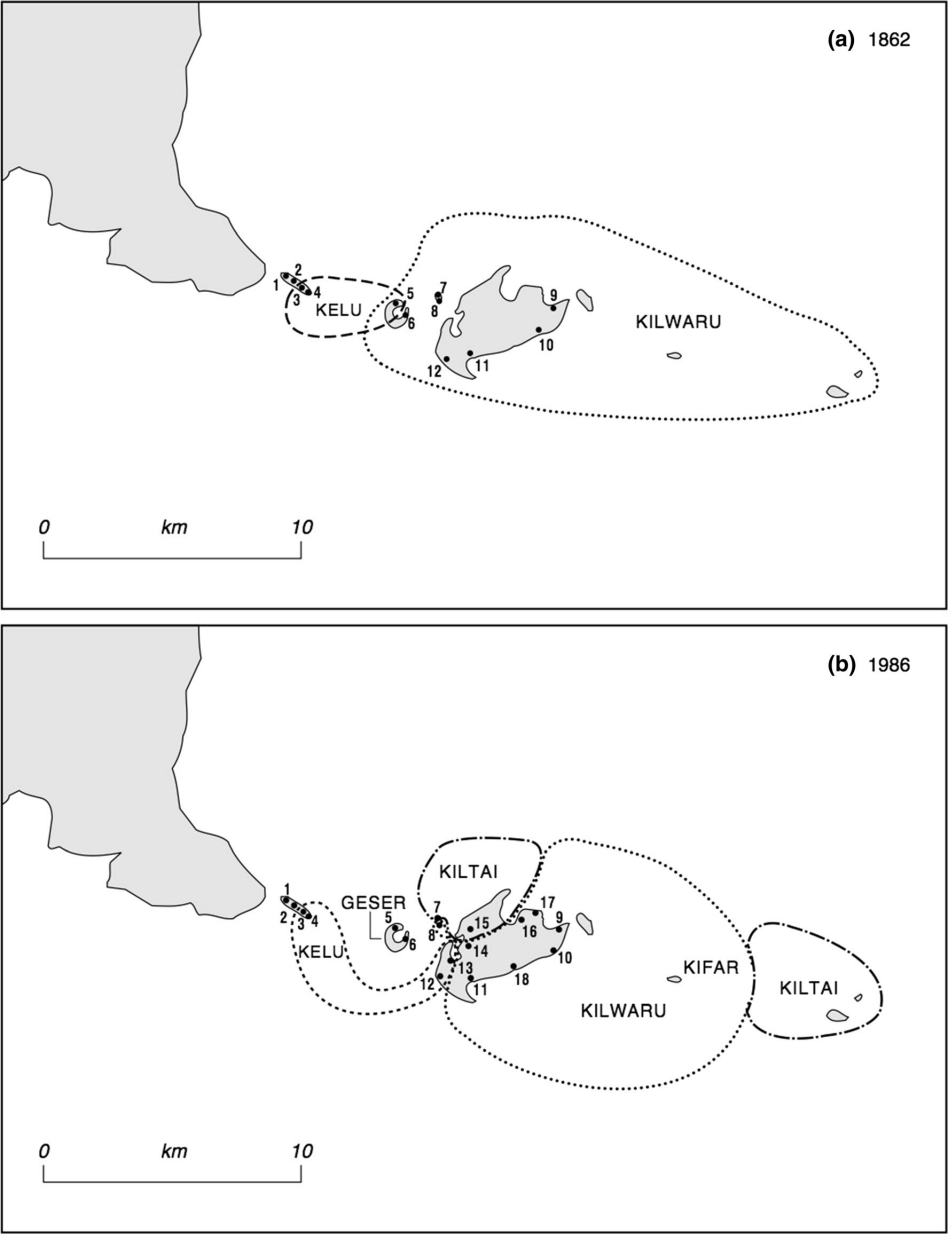
Changing domain boundaries in the vicinity of Geser: **a** 1862, and **b** 1986. *Source* Ellen 2003, p. 33

In understanding patterns of settlement and the resilience of the local system, we need to remember that pre-colonial Southeast Seram was not a single political entity, but rather comprised a number of independent local domains. These achieved the sustainability required for trade through overlapping sets of relations at different levels. Some domains were tiny, others cut across islands or joined together islands that were quite separate. For example, Garogos within the domain of Kataloka in 1986 had strong cross-cutting ties of marriage with Kidang in the domain of Kilwaru. Indeed, until domain boundaries stabilised under the Dutch at the end of the nineteenth century, these habitually moved in response to shifting political relations (Fig. 5). This allowed for access to different biotopes, fishing grounds, garden land, sago and forest within a domain, but some small island domains were entirely reliant on trade between domains, or arrangements for (say) cutting sago or firewood in other domains. At the highest level there were traditional political alliances (such as *Ulilima, Ulisiwa* and *Totu tolu*) linking often widely distributed domains that provided security and context for more mundane economic transactions and redistribution of resources, reinforced by symbolic and mytho-historical legitimation (Ellen 2003, pp. 34–35). Thus, social complexity supported greater environmental resilience.

## Sea-level rise and coral growth

In 1985, Geser Meteoreological station was reported as being 1.50 m above sea level. It is commonly assumed that once sea level rises above reef foundations, otherwise stable atolls and cays such Geser, Kiltai, Seram Rei and Garogos, will be subject to wave erosion and eventually ‘drown’ (Webb and Kench 2010). This model predicts that inhabitants will become climate or environment refugees (Yamamoto and Esteban 2014), and local terrestrial biodiversity extinct.

In the Geser–Gorom corridor, where perturbations are frequent, islanders have histories of moving to more stable places. The inhabitants have learnt to shift around to avoid short-term problems, just as dwellers on volcanic islands have moved to escape eruptions. Unfortunately, the physical and administrative infrastructures of modern states do not always have this flexibility, and quasi-urban settlements such as Geser, with a high concentration of cement houses with solid foundations, have lost this flexibility. The bigger, more frequent, storms associated with climate change, and the inevitable rigidity of response these changes engender may increase the likelihood of producing ‘environmental refugees’.

We have known since Darwin (Stoddart 1994) that coral islands are diverse biological systems growing in a particular way over time. Given the right conditions, coral reproduces fast enough to keep pace with subsidence predicted by sea-level rise. Thus, Kench et al. (2015), using data for the last 100 years, found no evidence of heightened erosion on oceanic islands studied and conclude that they had either grown or changed shape, storms and other disturbances likely churning the sea, breaking-up coral and depositing it on atolls better explaining stability than sea level. We can see these effects in the Geser–Gorom corridor.

Darwin was unaware of Quaternary changes in sea level leaving coral exposed to chemical weathering (Purdy and Winterer 2001); neither could he appreciate pollution effects and ocean warming. We now know atoll formation to be a fluctuating rather than continual process. Because reefs flourish in a narrow range of conditions, growth may slow as temperatures increase, are too low or water acidic, although new coral may re-establish along shallow shorelines. Atolls, by contrast, have solid foundations above sea level allowing accumulation of organic matter, increasing resistance to wave damage, and long-term stability. Dated artefacts from archaeological sites shows Pacific oceanic atolls to have been inhabited for 1000–1500 years (Dickinson 2009). We have no archaeology indicating first dates of occupation in the Geser–Gorom corridor, but we expect occupation earlier than in Micronesian and Polynesian sites, given geography and patterns of early migration into the Pacific (e.g. Lape 2003, p. 102).

Despite observations by Darwin and his successors, suggesting coral growth as a mitigating factor in sea-level rise, changing environments may still prevent reefs growing fast enough to prevent drowning (Grigg 1982). Once foundations submerge, atolls become vulnerable to wave damage, and erosion removing rubble, sediment and thin soils, making them uninhabitable. Moreover, acidification and bleaching, due to loss of symbiotic coral algae following temperature rise in surrounding shallow seas, may curtail or stop growth, reducing the rate at which reefs recover.

Coral islands are not passive systems. They respond dynamically in complex ways to climate changes (Tomascik et al. 1997, p. 608; Kench et al. 2018). Relative sea-level rise depends on many factors, including sediment supply relative to submergence, width of fringing reef, growth rates, degree of anchorage of islands to rock platforms, composition, the incorporation of features such as beach rock outcrops and mangroves protecting the shore, reef health, and especially tectonic history. High rates of tectonic uplift typical of the Banda Arc suggest sea-level rise to be less influential (Tomascik et al. 1997, p. 1229). Many mitigating factors are evident in the Geser–Gorom corridor. More significantly, however, the classic atoll-drowning scenario was modelled on oceanic islands rather than where atolls and banks are parts of complex mainland coastal systems, where additional forces are at work (ibid. 594).

## Discussion: biological consequences of multi-niche systems

What does all this mean for biodiversity and the role of humans in its fluctuation? We have in southeastern Seram what Kent Flannery (1972) called a ‘multi-niche system’ in which a variety of biotopes are connected through human exchange. The main settlements are mostly on small coral sandbanks and atolls. Vegetable foods and starch staples are supplied either through kinship access to land on Seram Laut or mainland Seram, or through trade and barter with mainland Seram and the larger Gorom islands. In addition, the hubs rely on specialist mangrove areas for firewood. While mangrove has eroded, historical evidence suggests that boundaries have altered little over 500 years. The continuity is more remarkable than the depletion. Adjacent mainland rainforest remains biodiversity rich, buffered from over-extraction through difficulties of access. In order to survive, inhabited islands, in addition to being hubs in regional trade, rely on biodiversity-rich warm shallow seas for the harvesting of fish for subsistence and local exchange, and mainly ‘lola’ (*Trochus niloticus)*, ‘batu laga’ (*Turbo marmoratus*), sea-cumber (‘teripang’) and shark fin for commercial trade, while dugong (*Dugong dugong*) graze on sea grass, but are only hunted opportunistically. The first two of these, dugong and three species of turtle are protected under Indonesian law, although green turtles are exported to Bali (Monk et al. 1997, pp. 576–599). Customary law institutions have some effect mitigating overharvesting (Soselisa 2004), but there has been overfishing of the commercially valuable alga ‘agar–agar’.

Furthermore, in assessing biodiversity fluctuation, we should include coral species themselves, sometimes peripheralised in social science studies, which emphasise more organisms for which coral is the habitat. In the Geser–Gorom corridor, there is a contradiction, surely repeated elsewhere—that while much living reef is still biodiversity rich, terrestrial biota is biodiversity poor. Whatever terrestrial biota once existed on these cays, atolls and banks, for a period of over 500 years they have been subjected to impact through high human population densities, depleting mangrove and damaging coral in the immediate vicinity. There is likely also degradation from wave action, storm damage, bleaching and possibly—temperature increase (Tomascik et al. 1997). Dead coral has repercussions for human livelihoods, both through reduction of habitat possibilities and trophic flows, but also because it no longer counters sea-level rise over the long term.

The data and arguments presented here are consistent with a pattern that has been demonstrated by others on how small island ecosystems have been fundamentally reshaped through declining non-anthropic biodiversity, invasive colonisation of non-native species, changes in physical landscape, seismic instability, coastal erosion and sea-level rise. Much of this degradation results from human activities, although resource management has been shown to enhance productivity and sustainability (Braje et al. 2017, Delgado et al. 2017). We now know that islands such as those in the Geser–Gorom corridor are far from isolated biologically, being dynamic and open systems, living organisms moving across their boundaries, and islands interacting with each other. The smaller the island, the more intense and frequent the disturbance, the more human activity exacerbates the influence of sea-level rise, extreme weather events (high tides and flooding), hurricanes, earthquakes and volcanism (Delgado et al. 2017, p. 373).

However, I am concerned less with decline in terrestrial and marine biological richness caused by trade and other factors, than with a structural re-alignment in the distribution of resources in a particular area. I have asked what the impacts of potential climate change might be, and conclude that while these small islands appear superficially fragile (low lying and subject to sea-level rise, vulnerable to sea incursions, with unreliable supplies of fresh water, poor in terrestrial resources, heavily reliant on trade to supplement subsistence), they are paradoxically a zone of robustness looked at more widely. Inter-island trade in a multi-niche system can re-organise patterns of biological interaction spatially and over the long term and mitigate species loss at the level of the whole system. Connectedness is crucial (Kelman 2017, pp. 249–250), while settlement viability, especially the nexus of exchange that supports settlements, depends on hydrography as much as topography, and how this has determined central places and facilitated the social integration of the trading zone.

## Conclusion

Outside the colonial and post-colonial mainstream, archipelagic southeast Seram has remained important in regional trade for centuries. Relations of exchange have integrated a complex environmental system, affording a degree of protection through local human interventions. The constraints are restricted land surfaces, a limited range of starch staples, endemic environmental hazards, and the uncertainties of marine extraction. Given this risk pattern, an inter-island economy and social system allowing flexibility and breadth of resource accessibility, and for periodic refuge, is a precondition for long-term sustainability. Physical geography (winds, currents, land distribution) and ecology have encouraged and moulded the development of a system of inter-island social ties. By integrating ethnographic, ecological and historical evidence, we find a very nuanced picture. As is common with all complex systems, it is impossible to make simple predictions, although it is likely that, as in the past, biocultural trade-offs established across the Geser–Gorom corridor will mitigate long-term climate-change effects in the immediate future. If sea-level rise is over a metre by 2100 (Geisler and Currens 2017), then the mid-term scenario for the archipelagic hub is much worse.
